# PD-L1 Blockade During Allergen Sensitization Inhibits the Synthesis of Specific Antibodies and Decreases Mast Cell Activation in a Murine Model of Active Cutaneous Anaphylaxis

**DOI:** 10.3389/fimmu.2021.655958

**Published:** 2021-04-22

**Authors:** Rafael Bonamichi-Santos, Marcelo Vivolo Aun, Jorge Kalil, Mariana Concepcion Castells, Pedro Giavina-Bianchi

**Affiliations:** ^1^ Clinical Immunology and Allergy Division, University of São Paulo School of Medicine, São Paulo, Brazil; ^2^ Division of Rheumatology, Immunology, and Allergy, Department of Medicine, Brigham and Women’s Hospital, Harvard Medical School, Boston, MA, United States; ^3^ Faculdade Israelita de Ciências da Saúde Albert Einstein, Hospital Israelita Albert Einstein, São Paulo, Brazil

**Keywords:** PD-L1, anaphylaxis, murine model, active cutaneous anaphylaxis, inhibition

## Abstract

Programmed cell death ligand 1(PDL-1) is known for its inhibitory effect on the cellular immune response. Even though it is expressed on the surface of mast cells, its role in allergic diseases is unknown. We analyzed the effects of PD-L1 blockade in a murine model of active cutaneous anaphylaxis (ACA). C57BL/6 mice were sensitized and challenged with ovalbumin (OVA). Blood samples were collected to measure specific immunoglobulins. The mice were divided into six groups that underwent the active cutaneous anaphylaxis procedure. Group 1 (negative control) received 50 μl of phosphate-buffered saline (PBS) subcutaneously, and the other five groups were sensitized with 50 μg of OVA subcutaneously. Group 2 was the positive control, and the others received the anti-PD-L1 antibody or its isotype during sensitization (groups 3 and 4) or during the challenge (groups 5 and 6). All animals that underwent ACA on the ears with OVA and PBS were sacrificed, and the reaction was evaluated by extravasation of Evans blue (measured by spectrophotometry) and histological analysis of the collected fragments. Anti-PD-L1 blockade during the sensitization phase led to a reduction in specific IgE and IgG1 levels, allergic reaction intensity at the ACA site, and mast cell degranulation in the tissue. There was no significant biological effect of anti-PD-L1 administration on the challenge phase. PD-L1 blockade during allergen sensitization inhibited the synthesis of specific IgE and IgG1 and decreased mast cell activation in this murine model of anaphylaxis.

## Introduction

Anaphylaxis is an immediate systemic hypersensitivity reaction induced by mast cell and basophil degranulation and is a medical emergency that can lead to death (1, 2). Studies have shown a growing incidence and mortality of anaphylactic reactions, especially those induced by drugs and food (2).

Animal models are essential to better understand the pathophysiological mechanisms involved in diseases and to evaluate the safety and efficacy of new therapies before starting clinical trials in humans. Anaphylaxis has been reproduced and analyzed in murine models thanks to the practicality of rearing, breeding, maintaining, and handling these animals and their availability, including knockout and transgenic models (3). Several animal models have been developed to study the mechanisms involved in allergic inflammation, including models of respiratory and food allergies and systemic and local anaphylaxis (3–8). Cutaneous anaphylaxis induced in animal models can be divided into active and passive anaphylaxis. In the active cutaneous anaphylaxis (ACA) model, mice are sensitized by receiving fractionated doses of the allergen, whereas in the passive cutaneous anaphylaxis (PCA) model, the animals are passively sensitized by receiving serum from other mice that were previously actively sensitized (9).

The levels of specific serum immunoglobulin E (IgE) and IgG1, which have anaphylactic functions in mice, and mast cell degranulation are often measured as markers of IgE-mediated allergic responses in animal models. Murine IgG1, present in the Th2 immune response, resembles human IgG4, and murine IgG2a, present in the Th1 immune response, has a similar function as human IgG1 (9). In the evaluation of mast cell degranulation, cells with preserved granules can be quantified by chloroacetate esterase (CAE) staining techniques, and spectrophotometric techniques are needed to measure the extravasation of dyes in tissues where there is increased vascular permeability (5, 10).

Recent studies have further clarified the factors that reduce the antitumor immune response, leading to the discovery of several molecules that act in the costimulatory and coinhibitory control pathways, called checkpoint pathways. A checkpoint pathway molecule that mediates tumor-induced immune suppression is the programmed cell death 1 (PD-1) protein. PD-1 is considered a member of the CD28 receptor family, while programmed cell death ligand 1 (PD-L1), one of its ligands, is a member of the B7 receptor family and is also known as CD274 (11, 12). PD-1 is expressed in the membrane of T and B lymphocytes, sending inhibitory signals into these cells when activated by its ligand PD-L1 or PD-L2, which are found in the membrane of dendritic cells and monocytes, but also in tumor cells (13, 14).

Physiologically, the PD-1/PD-L1 pathway works to control the degree of inflammation to prevent an exacerbated immune response with damage to normal tissue. There is marked expression of the PD-1 protein on the surface of activated T cells. When a T lymphocyte recognizes the antigen expressed by the MHC complex in the target cell, inflammatory cytokines are produced that induce the expression of PD-L1 in the tissue, which binds to and activates PD-1 in the T cell, inducing immune tolerance (15). In pathological processes, the activation of the PD-1 receptor by its ligands has an inhibitory effect on “exhausted” T lymphocytes against persistent chronic antigenic stimulation, as observed in tumors and chronic infections. The PD-1/PD-L1 pathway and its modulation are being widely studied in oncology, and the evolution of immunotherapy with antibodies against coinhibitory molecules in the treatment of cancer is one of the most successful therapeutic discoveries in recent years (16–18).

In addition to the inhibitory effect of PD-1 on lymphocytes, its binding to PD-L1 or PD-L2 leads to polarization of the immune response toward the Th2 or Th1 profile, respectively (19, 20). In an animal model of respiratory allergy, pulmonary dendritic cells express PD-L1 and PD-L2 after antigen recognition and activation. The PD-1/PD-L1 interaction produces a Th2 response with increased production of IL-4 and increased airway hyperresponsiveness (AHR). However, the PD-1/PD-L2 interaction initiates a Th1 response with increased expression of IFN-*γ* and, subsequently, a reduction in AHR (20).

A study in cultured mouse mast cells showed that these cells express several costimulatory and coinhibitory molecules in their membrane, including members of the B7 family, such as PD-L1 (21). Studies analyzing the expression of PD-L1 and its functions in human mast cells are lacking. Despite the many studies on the mechanisms of anaphylactic reactions, little is known about the coinhibitory pathway of the PD-1 receptor and its PD-L1 ligand in anaphylaxis. Exploring the coinhibitory pathway of immunoregulation in an anaphylaxis model could expand the range of treatment and prevention resources.

The objective of the present study was to evaluate the effects of blockade of the PD-L1 molecule on the sensitization and effector phases of ACA. We hypothesized that by blocking the PD-1/PD-L1 interaction, we could observe either an increase in the allergic reaction due to the lower inhibition of T lymphocytes, or even a decrease in the reaction due to weaker polarization of the immune response toward the Th2 profile.

## Materials and Methods

### Animals

A total of 30 adult C57BL/6 mice aged 6 to 8 weeks were used, provided by Jackson Laboratory (Bar Harbor, Maine), which were reared according to the guidelines of the National Institutes of Health (NIH). The project was approved by the ethics committee of the two institutions involved in the project, the Dana-Farber Cancer Institute, Boston, Massachusetts (DFCI IRB 15-046) and the University of São Paulo Medical School (CEUA-FMUSP 1286-2019).

### Experimental Design

The antigen was ovalbumin (OVA), and the adjuvant used was aluminum hydroxide, both from Sigma-Aldrich. The PD-L1 protein was blocked by the anti-PD-L1 antibody Ultra-LEAF™ Purified anti-mouse CD274 (B7-H1, PD-L1) (BioLegend). The Ultra-LEAF™ Purified Rat IgG2b antibody, κ isotype (BioLegend) was used as a control for the intervention that would not activate or block the PD-L1 molecule.

We used the local anaphylaxis technique to evaluate the allergic inflammatory reaction, more specifically the ACA technique, where the sensitization and challenge phases are performed in the same animal. The sensitization protocol lasted 28 days and was performed subcutaneously (sc) at the base of the mouse’s tail.

**Figure 1:**
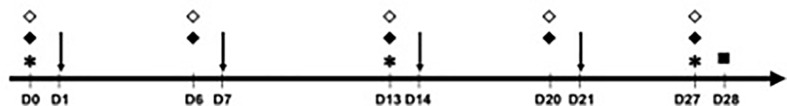
Timeline of the experimental protocol (29-day duration – from D0 to D28). Animals were sensitized with phosphate-buffered saline (PBS) or ovalbumin (OVA) on days 1, 7, 14 and 21 and challenged on D28, when euthanasia was also performed. * Blood collection for measurement of immunoglobulins (all groups); ↓ sensitization with PBS (group 1) or OVA (groups 2 to 6); ♦ anti-PD-L1 (during sensitization in group 3 or challenge in group 5); ⋄ anti-PD-L1 isotype (during sensitization in group 4 or challenge in group 6); ▫ euthanasia, active cutaneous anaphylaxis and skin biopsy (all groups).

The analyzed mice were divided into six groups of five animals (**Table 1**). The animals in group 1 (negative control) received injections of 50 μl of phosphate-buffered saline (PBS) in the sensitization phase, whereas the animals in the other groups were sensitized with 50 μl of OVA at 1 μg/μl on days (D) 1, 7, 14, and 21. To evaluate the effects of PD-L1 blockade in both the allergic sensitization and the effector (challenge) phase, four of these groups were administered anti-PD-L1 antibody (groups 3 and 5) or its anti-PD-L1 isotype (groups 4 and 6) intraperitoneally at a dose of 200 μl per application. These antibodies were administered one day before each OVA application (D0, D6, D13, and D20) in groups 3 and 4 to evaluate sensitization or one day before challenge (D27) in groups 5 and 6 to evaluate the effector phase. Thus, the six groups were the negative control (PBS), positive control (OVA), anti-PD-L1 in the sensitization phase, anti-PD-L1 isotype in the sensitization phase, anti-PD-L1 in the challenge phase, and anti-PD-L1 isotype in the challenge phase (**Table 1** and **Figure 1**).

**Table 1:** Experimental groups according to substances and techniques used.

Experimental groups	Technique	Sensitization	Challenge	Antibody	
				Sensitization	Challenge
1 – Negative control	ACA	PBS	PBS	-	-
2 – Positive control		OVA	OVA	-	-
3 – Anti-PD-L1 during sensitization				Anti-PD-L1	-
4 – Anti-PD-L1 isotype during sensitization				Isotype	-
5 – Anti-PD-L1 during challenge				-	Anti-PD-L1
6 – Anti-PD-L1 isotype during challenge				-	Isotype

PBS, phosphate-buffered saline; OVA, ovalbumin; ACA, active cutaneous anaphylaxis; Anti-PD-L1, Ultra-LEAF™ Purified anti-mouse CD274 (B7-H1, PD-L1) (BioLegend, ref.:124318); isotype, Ultra-LEAF™ purified rat IgG2b antibody, κ isotype (BioLegend, ref: 400644).

Blood samples were collected on D0, D13, and D27 from the ophthalmic plexus (300 μl/bleed) for measurement of specific IgE, IgG1, and IgG2a antibodies in plasma by indirect enzyme-linked immunosorbent assay (ELISA). On D28, the mice in group 1 (negative control) were challenged with 50 μl of PBS, and the animals in the other groups were injected with 10 μl of OVA at 5 μg/μl. On this occasion, mice from all groups also received 200 μl of 0.025% Evans blue intravenously. After 10 minutes, the animals were euthanized in a CO_2_ gas chamber, and the reaction was evaluated by the extravasation of Evans blue measured by spectrophotometry and by histological analysis of collected fragments (**Figure 1**).

### Measurement of Specific Antibodies

The specific IgE, IgG1, and IgG2a antibodies were measured in the plasma by indirect ELISA kits (Affymetrix and eBioscience). All five mice in each group had serum antibodies specific for OVA measured at D0, D14, and D28. The collected blood was centrifuged at 2000 rpm for 10 minutes, and the plasma was separated and frozen at -20°C. To quantify IgE, IgG1, and IgG2a, a microplate was coated with ovalbumin. After incubation and washing, the sera were added at a predetermined dilution. To develop the reaction, biotinylated detection antibody specific for IgE, IgG1, or IgG2a was added, followed by incubation and washing. Lastly, the developer solution containing streptavidin-peroxidase enzyme conjugate, substrate, and chromogen was added. The colorimetric reaction was read in a spectrophotometer at 450 nm. The results are expressed as mean absorbances and were compared with the standard provided by the kit in serial dilutions.

### Active Cutaneous Anaphylaxis Reaction

**Figure 2:**
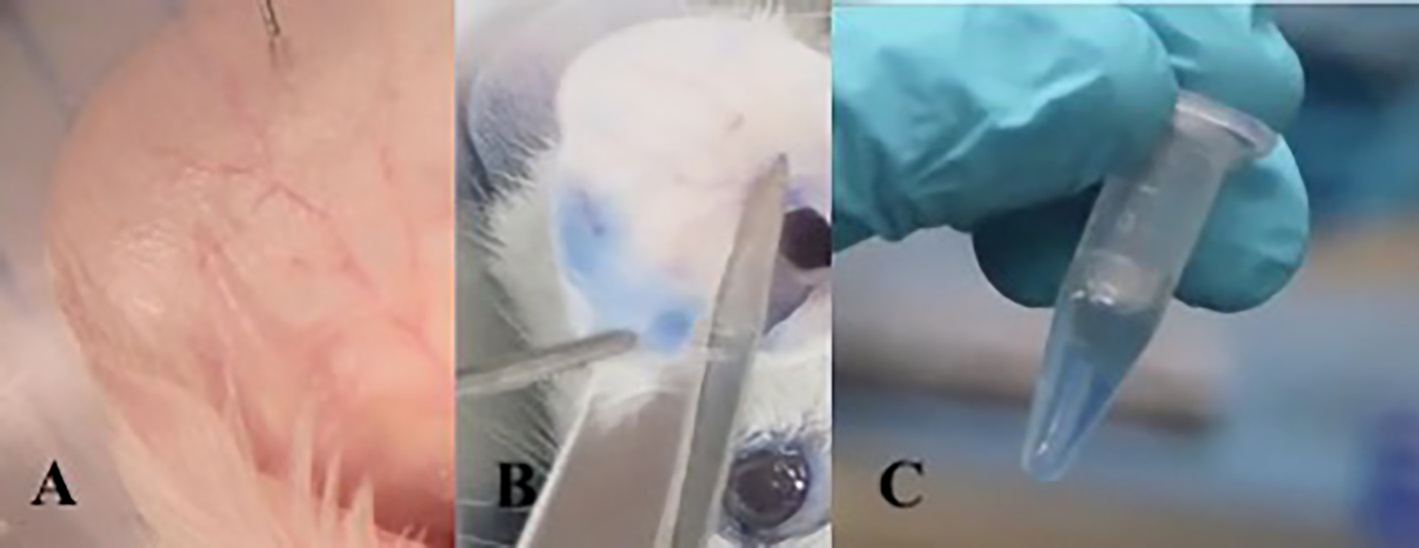
Active cutaneous anaphylaxis technique: Intradermal injection into the ear with allergen diluted in PBS. **(A)** After 10 minutes of exposure to the allergen, the mouse is sacrificed and the ear is sectioned. **(B)** The ear fragment is kept in formamide at 63°C for 18 hours to extract Evans blue dye from the tissue **(C)**.

The ACA assay was performed to evaluate the presence of a specific IgE with anaphylactic activity on the skin of sensitized mice. The animals received 10 μl of OVA at 5 μg/μl sc in the right ear. In the left ear, 10 μl of PBS was applied as a negative control. Then 0.25% Evans blue was administered intravenously. After 10 minutes of reaction, the animals were sacrificed in a CO_2_ chamber. The Evans blue that extravasated into the tissue was extracted after 12 hours in 700 μl of formamide at 63°C. Then, the absorbance of the solution composed of formamide and Evans blue was measured on a spectrophotometer at 620 nm. ACA was considered positive when the absorbance was greater than 0.150 nm (**Figure 2**).

### Histological Analysis: Staining and Quantification of Mast Cells

CAE, which stains the granules of mast cells, was used to stain the tissues. The number of stained cells is lower when these cells are activated because degranulation occurs in this process. Tissue samples from the ear challenged with OVA were fixed in 10% buffered formalin and embedded in paraffin, ensuring a transverse orientation of all tissues, and then cut to a thickness of 4 mm. The slides were scanned using a Pannoramic 250 Flash II 3D scanner (Histech, Budapest, Hungary). The mast cells were quantified according to area (mm^2^) using computer-generated image analysis (NIH ImageJ software, version 1.49v).

### Statistical Analysis

Significant differences between the experimental groups were detected by one-way analysis of variance followed by the nonparametric Mann-Whitney test or the parametric Bonferroni test. P-values < 0.05 were considered significant. SPSS software was used for statistical analysis.

## Results

### Specific IgE, IgG1, and IgG2a Antibodies With PD-L1 Blockade

**Figure 3:**
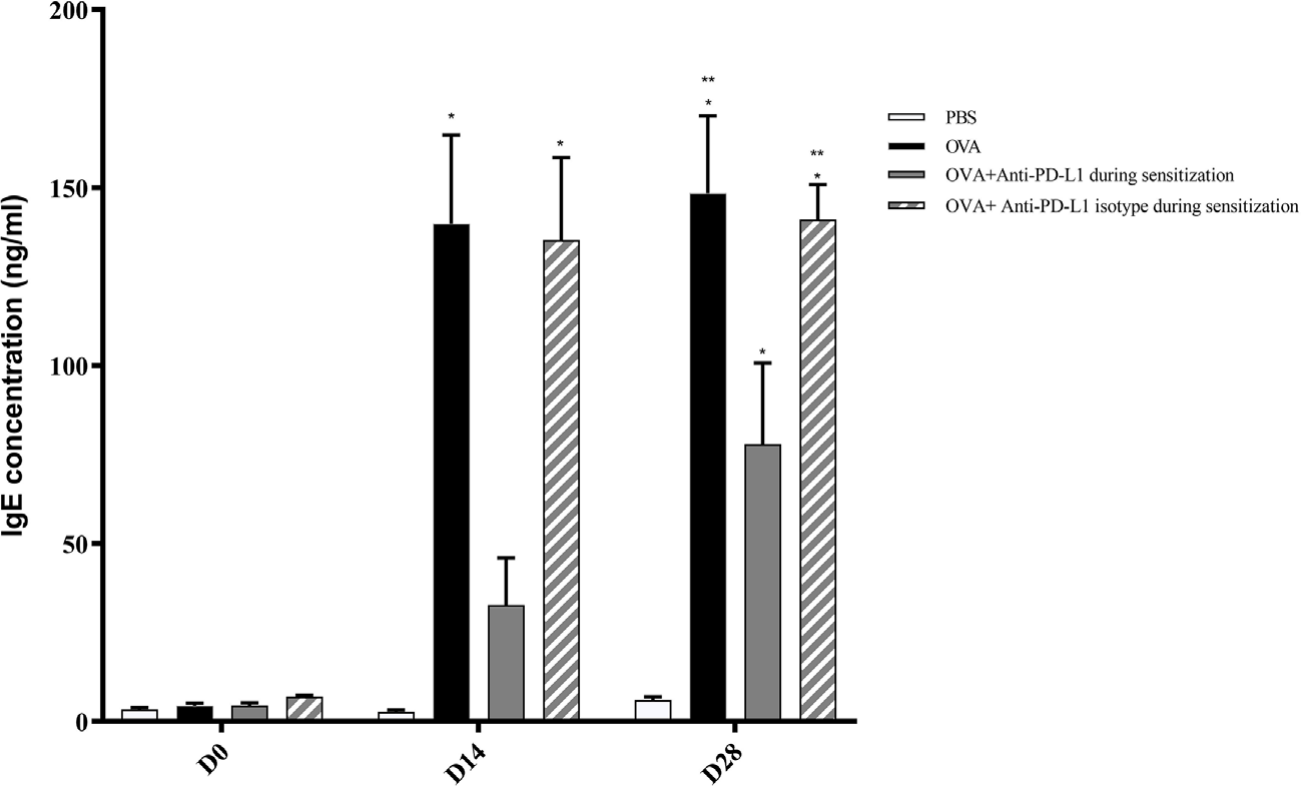
Concentration of serum anti-OVA IgE in groups 1, 2, 3, and 4. There was an increase in anti-OVA IgE in groups 2 and 4 on D13 and D27 and in group 3 on D27. Group 3 (anti-PD-L1) had lower anti-OVA IgE than groups 2 and 4. ★ p < 0.001 compared to group 1 (PBS); ★★ p < 0.001 compared to group 3 (anti-PD-L1). PBS, phosphate-buffered saline; OVA, ovalbumin.

Positive control and anti-PD-L1 isotype groups developed high levels of specific IgE for OVA since D13. At that moment, specific IgE from anti-PD-L1 group was comparable to control group (p>0.05). However, specific IgE levels from anti-PD-L1 group increased in D27, reaching higher levels than the negative control group, although they were lower than the positive control and anti-PD-L1 isotype groups (**Figure 3**).

**Figure 4:**
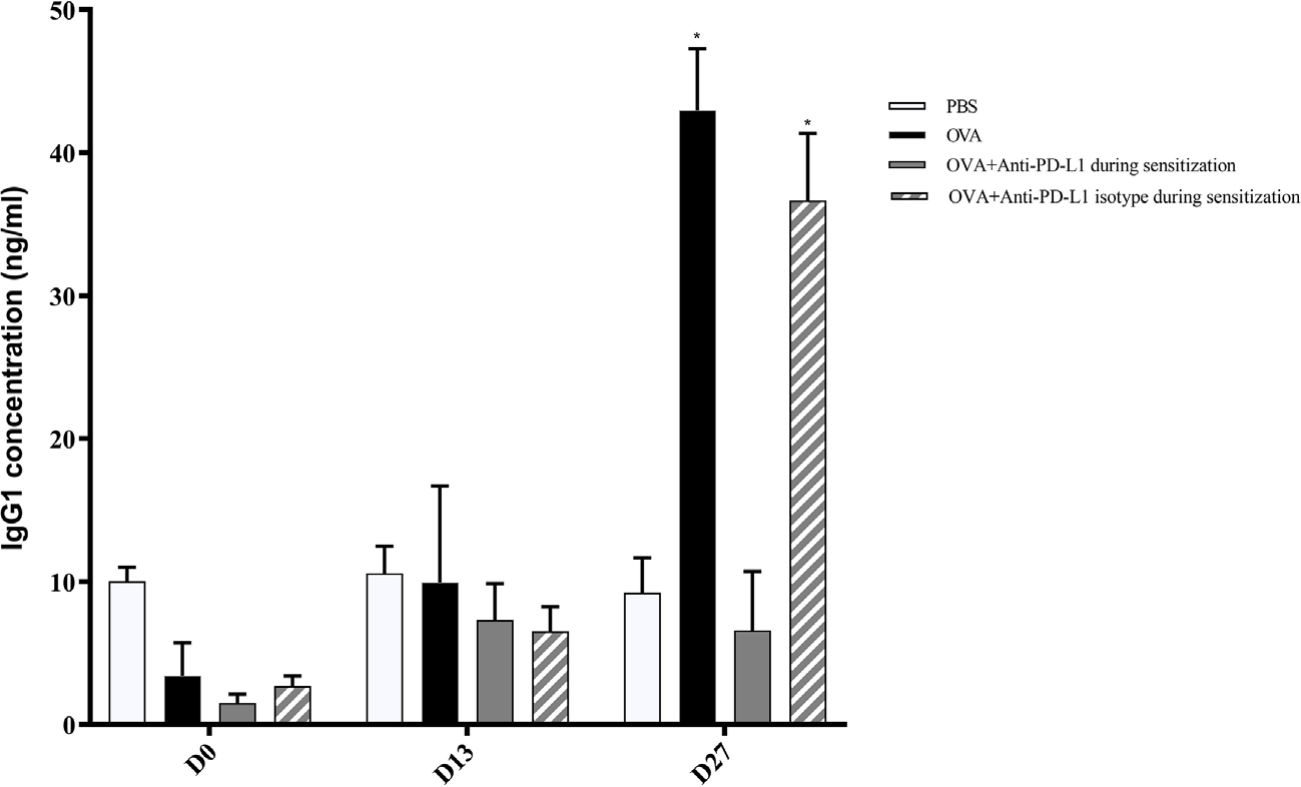
Serum anti-OVA IgG1 concentration in groups 1, 2, 3, and 4. There was an increase in anti-OVA IgG1 in groups 2 and 4 on D27. ★ p < 0.001 compared to group 1 (PBS). PBS, phosphate-buffered saline; OVA, ovalbumin.

Regarding specific IgG1, levels on D13 were still low and comparable between all groups. Nonetheless, serum specific IgG1 levels increased on D27 in the positive control and anti-PD-L1 isotype groups and this effect was blocked by anti-PD-L1 administration (**Figure 4**).

**Figure 5:**
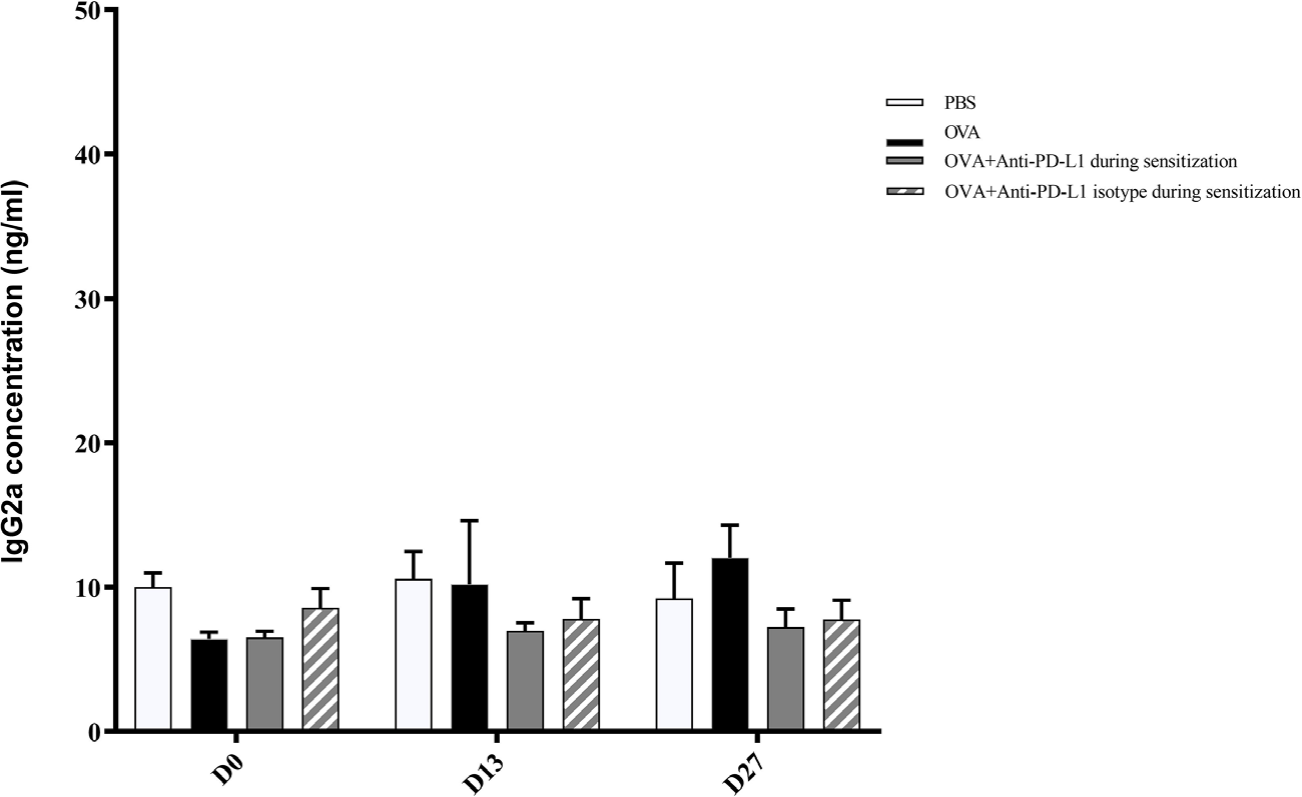
Serum anti-OVA IgG2a concentration in groups 1, 2, 3, and 4. There was no difference in anti-OVA IgG2a between groups. PBS, phosphate-buffered saline; OVA, ovalbumin.

There was no difference in the serum IgG2a level between the four groups (**Figure 5**).

### ACA With PD-L1 Blockade

**Figure 6:**
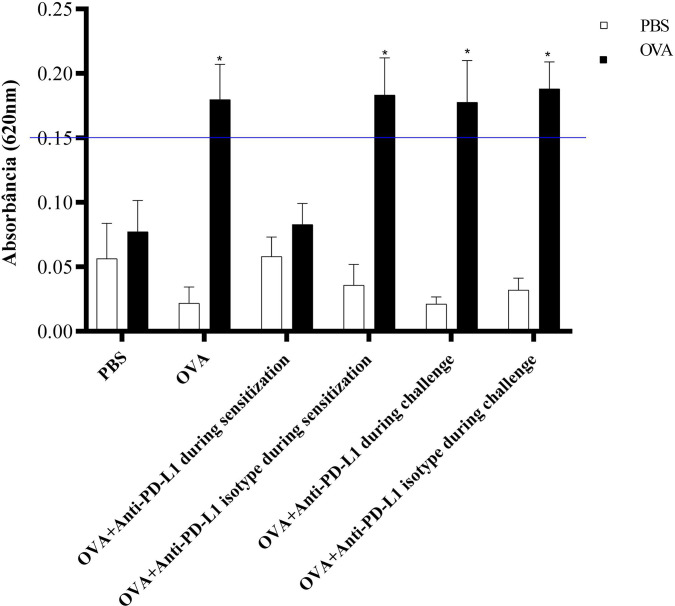
Absorbance of Evans blue extravasation in active cutaneous anaphylaxis (ACA) of the six experimental groups. All animals underwent the ACA test in both ears. PBS was administered to the left ear (negative control – white column) and OVA to the right ear (allergen – black column). There was an increase in extravasation in the right ear of groups 2, 4, 5, and 6. There was no difference between the PBS and anti-PD-L1 groups during sensitization. ★ p < 0.001 compared to group 1 (PBS). PBS, phosphate-buffered saline; OVA, ovalbumin.

The ACA reaction was considered positive, with significant extravasation of Evans blue, in groups 2 (OVA), 4 (anti-PD-L1 isotype during sensitization), 5 (anti-PD-L1 during challenge), and 6 (anti-PD-L1 isotype during challenge). Administration of the antibody anti-PD-L1 administered during sensitization (group 3) lead to inhibition of ACA, as shown in **Figure 6**.

### Histology With PD-L1 Blockade

**Figure 7:**
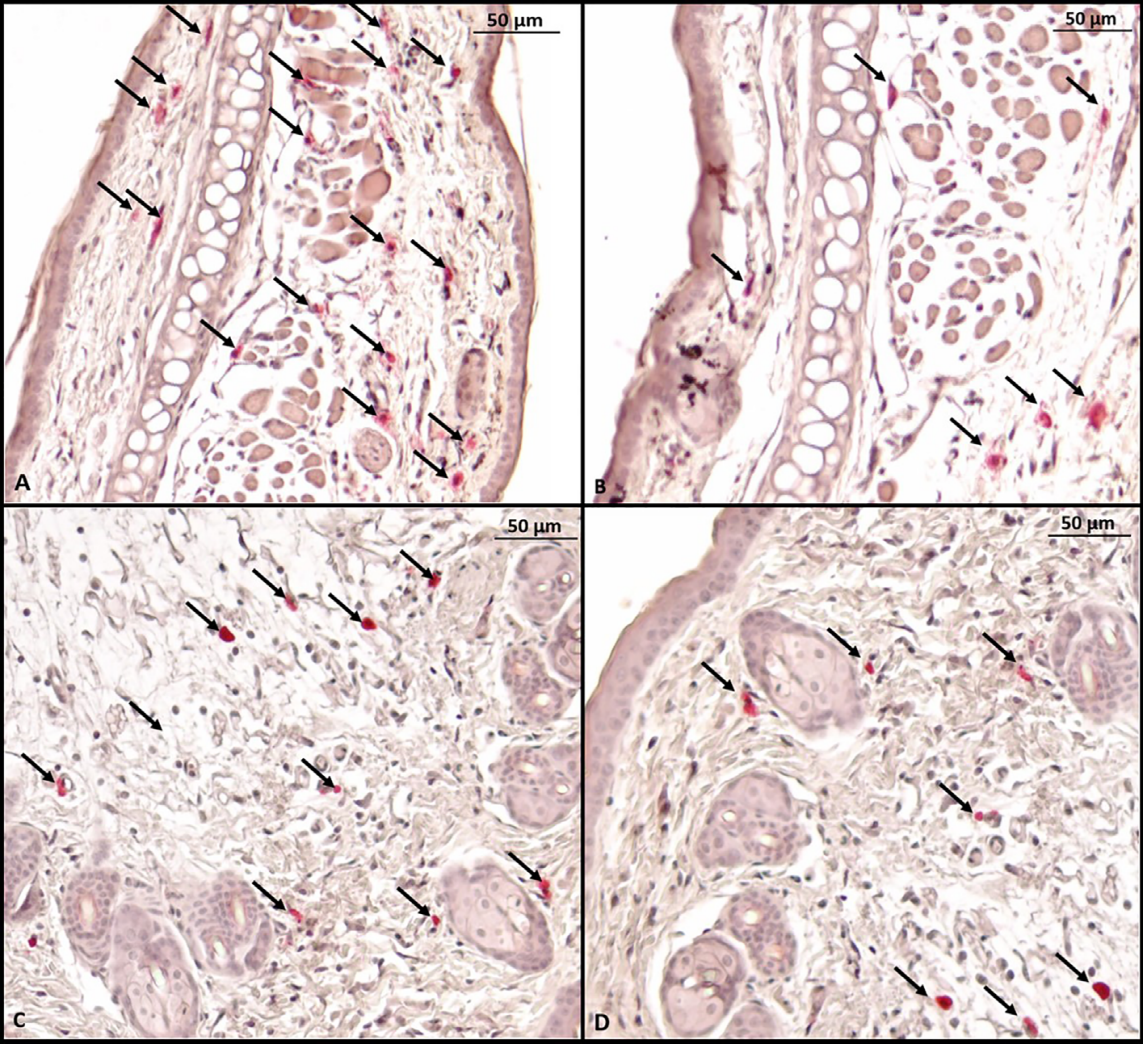
Microscopy of a mouse ear fragment stained with chloroacetate esterase. Mast cells are indicated by black arrows. **(A)** Group 1 (phosphate-buffered saline); **(B)** Group 2 (Ovalbumine); **(C)** Group 3 (anti-PD-L1); **(D)** Group 4 (anti-PD-L1 isotype).

**Figure 8:**
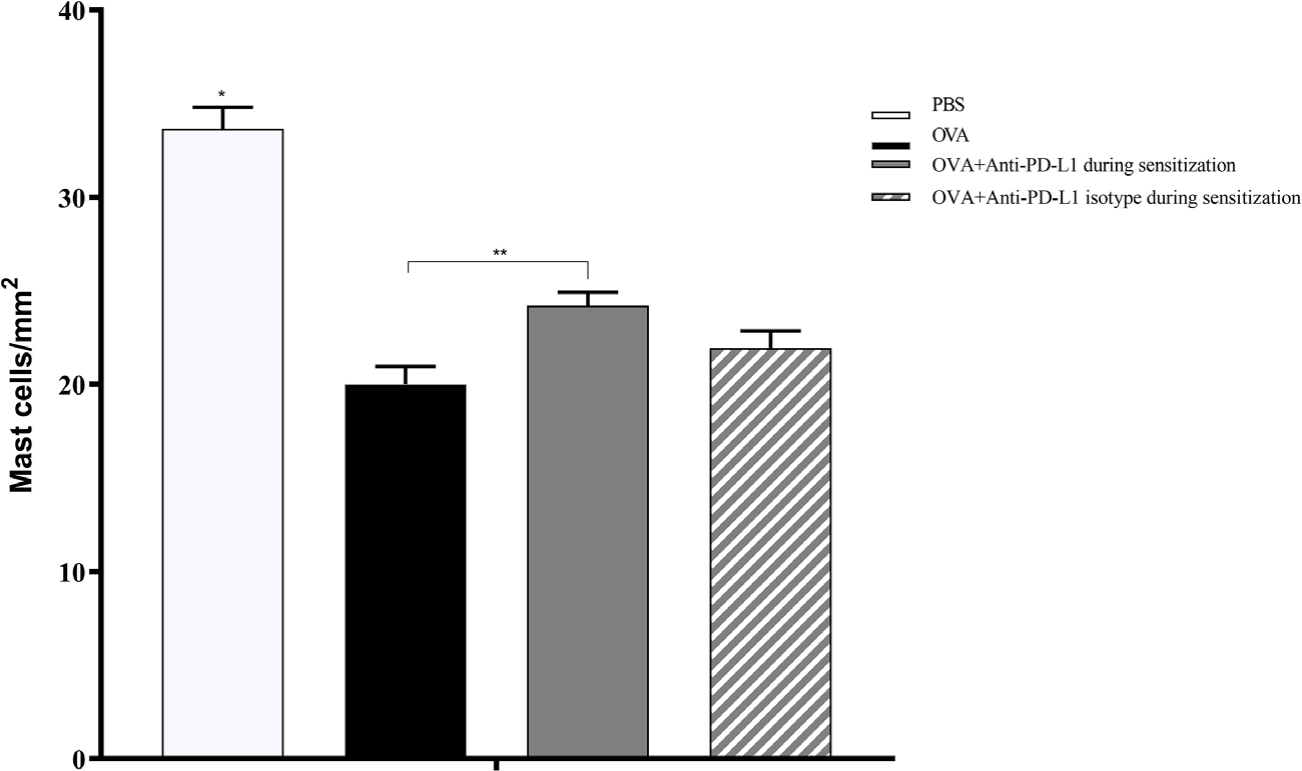
Histological analysis with the number of mast cells stained with chloroacetate esterase in groups 1 to 4. The number of mast cells was higher in group 1 (PBS) than in the other groups, suggesting lower degranulation. Group 3 (anti-PD-L1) had a higher number of mast cells than group 2 (OVA). ★ p < 0.0001 compared to the other groups; ★★ p < 0.01 between groups 2 and 3. PBS, phosphate-buffered saline; OVA, ovalbumin.

Histological evaluation was performed on two mice from each group. CAE staining of the tissue made it possible to visualize in red the granules of mast cells that were at rest, i.e., that had not degranulated. There were fewer tissue mast cells in the groups sensitized to OVA, but anti-PD-L1 administered at sensitization attenuated this effect (**Figures 7** and **8**).

## Discussion

Our study suggests that PD-L1 plays a crucial role in the activation of the Th2 immune response profile. In an animal model of OVA-induced allergy, PD-L1 blockade by anti-PD-L1 monoclonal antibody during sensitization decreased the specific immunoglobulins IgE and IgG1, as well as mast cell activation, as confirmed by ACA and by histology at the site of challenge. These effects were not observed when the blockade occurred only in the effector phase, suggesting that this pathway acts in the activation of Th2 cells and not directly in the activation of mast cells.

The PD-L1 receptor is strongly expressed in murine mast cells, but studies on its role in allergic processes are rare. To the best of our knowledge, this is the first study to evaluate the action of this protein in an allergy model focused on the immediate phase of the Gell & Coombs type I hypersensitivity reaction.

Some studies have attempted to elucidate the role of PD-1 pathways, induced by its binding to both PD-L1 and PD-L2, in murine models of allergic respiratory disease (19, 22–24). In a murine model of asthma, it was shown that PD-L1 was constitutively expressed in dendritic cells, macrophages, and B and T cells in the lungs of the animals and that this expression increased after challenge with ovalbumin. In turn, PD-L2 was poorly expressed in naïve dendritic cells, with a substantial increase after challenge. PD-L2 blockade at the time of allergen challenge, but not at sensitization, increased AHR and eosinophilia, in addition to IL-5 and IL-13 production, but reduced IFN-*γ* production in the lungs and lymph nodes. These effects were not observed with PD-L1 blockade, suggesting a particular role for PD-L2 in the asthmatic response (22). In contrast, in a study assessing the impact of manipulation of the PD-L1 and PD-L2 pathways on the development of asthma, it was reported that blockade of the PD-L2 pathway led to increased airway inflammation, IL-4, and AHR. Blockade of the PD-L1 pathway led to a reduction in AHR and increased the production of IFN-γ (19). In another study, using an animal model of allergic conjunctivitis, PD-L2 antagonism in the induction phase led to increased Th2 cytokine levels in the supernatant of splenocytes in culture. In addition, anti-PD-L2 in the effector phase led to increased influx of eosinophils into the conjunctiva of the animals. These effects were not clearly seen with PD-1 blockade (23).

Activation by the PD-L1, but not PD-L2, pathway can induce Foxp3^+^ regulatory T cells (25). The PD-1 pathway is indeed important for the action of Treg cells, as shown in a cockroach-induced asthma model. PD-1 blockade causes an increase in IL-4, IL-5 and IL-13, and a reduction in IL-10 in the bronchoalveolar lavage of challenged animals that have been subjected to Treg administration in the lungs (24). In a review article, based mainly on respiratory allergy models, the authors concluded that the PD-1/PD-L1 interaction seems to induce a Th2 response, with an increase in IL-4, whereas the PD-1/PD-L2 interaction induces a Th1 response with upregulated IFN-γ. Thus, it was suggested that the simultaneous expression of the PD-L1 and PD-L2 ligands could neutralize these effects and not cause any polarization (20).

However, in a more recent study in which different mice strains were subjected to another asthma model, blockade of the PD-1/PD-L1 pathway resulted in increased AHR, not by increasing Th2 activation but by increasing Th17. There were also varied effects on the different populations of helper T cells, and this effect also varied according to the mouse strain (26). We have not found experimental models in which the PD-L1/PD-L2 pathways have been evaluated in other allergic diseases mediated by IgE, such as systemic or cutaneous anaphylaxis.

In the present study, we blocked the interaction of PD-1/PD-L1 with anti-PD-L1, which may have resulted in weaker polarization toward Th2, with lower production of IgE and IgG1. The serum level of specific IgE impacts mast cell sensitization, explaining the observation of a lower number of degranulated mast cells and lower allergic reaction in the group treated with anti-PD-L1. Nonetheless, impact of anti-PD-L1 in specific IgE levels on D27 was lower than on D13, suggesting a partial failure on the effect of this monoclonal antibody with repeated injections. It is possible that higher doses may be necessary in long-term treatment with repeated injections of anti-PD-L1.

Previous studies have led to interest in evaluating the role of these pathways in allergic diseases in humans. Recently, it was shown that in adults with mild asthma subjected to segmental bronchial allergen challenge, there is an increase in the expression of PD-1 and PD-L1 24 hours after the challenge, corroborating the role of this pathway in this Th2-profile disease (27). Increased expression of PD-1 in the cell membrane of a patient with chronic rhinosinusitis with nasal polyposis (28) and of both PD-1 and PD-L1 in individuals with allergic rhinitis (29) have also been recently demonstrated.

The PD-1/PD-L1 pathway is classically associated with the modulation of the immune response of T cells, so blocking this pathway has become a standard strategy for the treatment of some types of cancer. Contrary to the findings in murine models, it was suggested that PD-L1 blockade in cancer in humans could aggravate pre-existing allergic diseases. A recently published clinical case of a patient with lung cancer and probable asthma–chronic obstructive pulmonary syndrome overlap syndrome reported that this patient presented worsened lung condition when subjected to immunotherapy with anti-PD-L1 durvalumab. However, there was no evidence of worsened pulmonary function or increased in eosinophils, making it difficult to confirm that the monoclonal antibody caused the exacerbation of the allergic condition (30). We can speculate that blockade of this immunomodulatory pathway could result in reduced regulatory cell function, aggravating pre-existing inflammatory diseases. An active search for regulatory T cell induction is ongoing through allergen-specific immunotherapy for allergic diseases (31). A recent clinical trial with patients allergic to peach subjected to sublingual immunotherapy showed that the expression of PD-L1 by peripheral-blood monocytes increased in the treated group, suggesting a role for this pathway in allergen tolerance induction (32). This suggests that immunotolerance may also use the PD-L1 pathway, but the mechanisms have not been fully elucidated.

Our study has some limitations. We used a small number of animals for histological analysis, which may have biased the results. Moreover, although unlikely, it is possible that PD-L1 pathway has some direct action in mast cell activation independently of the sensitization phase that was not investigated. Future studies could evaluate the role of PD-L1 in the effector phase in a PCA model, administrating it to animals that were not previously sensitized with the allergen. In addition, although we performed ACA in both ears, only the ear that received the OVA challenge was biopsied. Although unlikely, it is possible that there was some variation in the density of mast cells in the tissue challenged with PBS.

Although stimulation of the PD-1/PD-L1 regulatory axis has the paradoxical functions of inhibiting the immune response and inducing the Th2 immune response, in our study the PD-L1 pathway was directly associated with the allergic response. Its blockade inhibited the synthesis of specific IgE and IgG1, mast cell degranulation, and vascular permeability, which suggests that the induction of the Th2 immune response by PD-L1 exceeded the inhibitory action of this receptor on T cells. The immunomodulation of this axis may represent a new preventive and therapeutic option in the treatment of allergic diseases such as anaphylaxis.

## Data Availability Statement

The original contributions presented in the study are included in the article/supplementary material. Further inquiries can be directed to the corresponding author.

## Ethics Statement

The animal study was reviewed and approved by Dana-Farber Cancer Institute, Boston, Massachusetts (DFCI IRB 15-046)/University of São Paulo Medical School (CEUA-FMUSP 1286-2019).

## Author Contributions

RB-S, JK, and PG-B initiated and coordinated the project. MA, MC, and PG-B designed the experiments. RB-S and MA conducted the experiments. All authors analyzed the data. RB-S, MA, and PG-B wrote the manuscript. All authors contributed to the article and approved the submitted version.

## Funding

RB-S received a grant/scholarship for the research (Programa de Doutorado Sanduíche no Exterior) from Coordenação de Aperfeiçoamento de Pessoal de Nível Superior (CAPES), number 88881.135960/2016-1. This work was supported by the Instituto Nacional de Ciência e Tecnologia (INCT) and Conselho Nacional de Desenvolvimento Científico e Tecnológico (CNPq), number 465.434/2014-2.

## Conflict of Interest

The authors declare that the research was conducted in the absence of any commercial or financial relationships that could be construed as a potential conflict of interest.
